# Mustard-Induced Anaphylaxis in a 23-Month-Old Male: An Underrecognized Pediatric Food Allergen in the United States

**DOI:** 10.7759/cureus.114458

**Published:** 2026-08-13

**Authors:** Joshua A Katz, Saloni S Joshi, Sunil N Joshi

**Affiliations:** 1 Allergy and Immunology, Family Allergy and Asthma Consultants, Jacksonville, USA; 2 Health Sciences, University of Central Florida, Orlando, USA; 3 Health and Human Sciences, Florida State University, Tallahassee, USA; 4 Allergy and Immunology, University of Florida College of Medicine - Jacksonville, Jacksonville, USA

**Keywords:** allergic reaction, anaphylaxis, food-induced anaphylaxis, mustard allergy, mustard anaphylaxis, pediatric allergy

## Abstract

Mustard allergy is an uncommon but clinically significant cause of immunoglobulin E (IgE)-mediated food allergy that remains underrecognized in the United States despite its established allergenic potential in European pediatric populations. Mustard is frequently present as a hidden ingredient in condiments, sauces, and processed foods and is not currently required to be disclosed as a major allergen under the United States Food Allergen Labeling and Consumer Protection Act (FALCPA). We report a case of mustard-induced anaphylaxis in a 23-month-old male baby with a history of IgE-mediated egg allergy who developed diffuse urticaria, lip swelling, circumoral discoloration, and cough within minutes of isolated mustard exposure. Symptoms rapidly resolved following intramuscular epinephrine administration. Skin prick testing demonstrated marked mustard sensitization with a 15 mm wheal and 25 mm flare response. Prior pediatric studies have demonstrated that mustard allergy may present early in childhood and can provoke severe IgE-mediated reactions, including anaphylaxis. This case underscores mustard as an underrecognized trigger of pediatric food-induced anaphylaxis in the United States, highlights an important gap in current mandatory allergen labeling policy, and emphasizes the importance of maintaining clinical suspicion for uncommon food allergens in young children presenting with food-triggered systemic reactions.

## Introduction

The prevalence of food allergy among children has increased substantially over recent decades, representing a growing public health concern in the United States and worldwide. Current estimates suggest that approximately 7.6% of children in the United States are affected by food allergy, with the highest prevalence observed among allergens such as peanut, milk, egg, tree nut, and shellfish [1]. Food allergies are associated with significant morbidity, risk of anaphylaxis, reduced quality of life, and increasing healthcare utilization. Clinical practice guidelines from the National Institute of Allergy and Infectious Diseases (NIAID) and the American Academy of Allergy, Asthma and Immunology emphasize the importance of early recognition and prompt management of immunoglobulin E (IgE)-mediated food reactions because of the potential for life-threatening systemic involvement [2,3].

While most pediatric food allergy literature focuses on commonly recognized allergens, less common food triggers remain comparatively underrecognized despite their potential to cause severe allergic reactions. One such allergen is mustard, a spice widely incorporated into condiments, sauces, dressings, and processed foods. Mustard allergy is considered relatively uncommon in the United States; however, European studies have identified mustard as a clinically significant pediatric allergen capable of provoking severe IgE-mediated reactions, including anaphylaxis [4-6]. Unlike the European Union and Canada, where mustard is classified among mandatory labeled allergens, mustard is not currently included among allergens requiring mandatory disclosure under the Food Allergen Labeling and Consumer Protection Act (FALCPA). FALCPA currently covers nine major food allergens defined by federal law: milk, eggs, fish, crustacean shellfish, tree nuts, peanuts, wheat, soybeans, and sesame. This regulatory discrepancy may increase the risk of inadvertent exposure, particularly given mustard's frequent presence as a hidden ingredient in processed foods and spice mixtures [4].

Several European investigations have contributed substantially to the current understanding of mustard allergy in children. Rancé et al. demonstrated that symptoms frequently began before three years of age, with urticaria, angioedema, and atopic dermatitis among the most common clinical manifestations [6]. Morisset et al. later confirmed that mustard can provoke clinically significant allergic reactions in sensitized individuals through double-blind placebo-controlled food challenge testing [5]. Additional studies have demonstrated cross-reactivity between mustard and other members of the Brassicaceae family, including cabbage, broccoli, cauliflower, and turnip rape, as well as associations with mugwort pollen sensitization [7,8]. Despite these findings, published literature describing mustard-induced anaphylaxis in very young children within the United States remains limited. We report this case to add to the sparse North American pediatric literature and to highlight mustard as a clinically important, underrecognized allergen warranting increased awareness among U.S. allergists and pediatricians.

## Case presentation

A 23-month-old male baby with a history of IgE-mediated egg allergy presented to an outpatient allergy and immunology clinic for evaluation following a suspected mustard-induced allergic reaction. The patient was born full term without significant perinatal complications and had no history of asthma or chronic pulmonary disease. Prior allergy evaluation included negative skin prick testing to peanut and almond, which were subsequently introduced without complication. Family history was notable for atopic disease, including fish and tree nut allergy in a maternal aunt.

Approximately one month prior to presentation, the patient was eating outdoors when Heinz regular yellow mustard came into direct contact with his mouth while eating a hot dog. According to the family, the patient did not ingest the hot dog or bun itself, and mustard represented the only novel exposure contacting the oral mucosa. Importantly, the patient had previously tolerated hot dogs without adverse reaction, and the implicated mustard product did not contain egg ingredients. Within minutes of exposure, he developed diffuse blotchy urticaria involving the face, chest, and lower extremities accompanied by cough, facial flushing, lip swelling, and circumoral discoloration described by the parents as "purple lips" (Table 1). No vomiting, diarrhea, syncope, or seizure-like activity was reported.

**Table 1:** Clinical Timeline of Mustard-Induced Anaphylaxis

Time/Event	Clinical Findings
Prior history	IImmunoglobulin E (IgE)-mediated egg allergy; tolerated hot dogs previously
Mustard exposure	Heinz yellow mustard contact while eating a hot dog; no other novel exposures
Minutes after exposure	Diffuse urticaria, lip swelling, facial flushing, cough, circumoral discoloration
Acute treatment	Intramuscular epinephrine (EpiPen Jr 0.15 mg) administered; rapid symptom resolution
Emergency evaluation	Diphenhydramine and observation; no biphasic reaction
Allergy follow-up	Mustard skin prick testing: 15 mm wheal/25 mm flare; egg white: 6 mm wheal/10 mm flare
Final diagnosis	IgE-mediated mustard-induced anaphylaxis

The constellation of acute mucocutaneous and respiratory symptoms met the established National Institute of Allergy and Infectious Diseases/Food Allergy and Anaphylaxis Network (NIAID/FAAN) clinical criteria for anaphylaxis [2]. Intramuscular epinephrine (EpiPen Jr 0.15 mg; Viatris, Canonsburg, PA) was administered promptly with rapid improvement in respiratory symptoms and rash. The patient was subsequently evaluated in the emergency department, where he received dirphenhydramine and observation prior to discharge. No biphasic reaction was reported.

At outpatient follow-up evaluation, physical examination demonstrated a well-appearing child in no acute distress with normal cardiopulmonary findings and oxygen saturation of 98% on room air. Percutaneous skin prick testing using mustard extract demonstrated marked sensitization with a 15 mm wheal and 25 mm flare response. Concurrent testing to egg white revealed a comparatively smaller 6 mm wheal and 10 mm flare response (Table 2).

**Table 2:** Skin Prick Testing Results

Allergen	Wheal Size	Flare Size	Interpretation
Mustard extract	15 mm	25 mm	Strong positive
Egg white	6 mm	10 mm	Positive sensitization

Given the patient's immediate systemic symptoms following isolated mustard exposure, rapid clinical response to epinephrine, prior tolerance of the accompanying foods, and strong confirmatory skin prick testing, a diagnosis of IgE-mediated mustard-induced anaphylaxis was established. Serum mustard-specific IgE testing was deferred at the time of initial evaluation given the unambiguous clinical presentation and is planned at future follow-up. An oral food challenge was not pursued given the severity of the index reaction; the clinical presentation independently satisfied the established NIAID/FAAN diagnostic criteria for anaphylaxis without challenge confirmation [2,3]. The family was counseled extensively regarding strict mustard avoidance, recognition of hidden mustard-containing foods, emergency anaphylaxis management, and continued carriage of epinephrine auto-injectors. Long-term follow-up was planned for longitudinal reassessment, including consideration of serum mustard-specific IgE testing and evaluation of natural history.

## Discussion

Mustard allergy remains an uncommon but clinically significant cause of IgE-mediated food allergy, particularly in pediatric populations. While the prevalence of food allergy in U.S. children continues to increase overall, most literature and public awareness efforts focus on common allergens such as peanut, milk, egg, and tree nuts [4-8]. In contrast, mustard remains comparatively underrecognized in the United States despite its established allergenic potential in European populations. Sharma et al. described mustard as an important hidden allergen because of its widespread incorporation into condiments, sauces, dressings, spice blends, and processed foods, creating an increased risk for inadvertent exposure [4].

A narrative literature review was performed using PubMed and Google Scholar utilizing combinations of the search terms "mustard allergy," "mustard anaphylaxis," "pediatric mustard allergy," "mustard allergy toddler," and "mustard allergy case report." Available literature regarding mustard allergy was found to originate predominantly from European cohorts, with limited published data describing mustard-induced anaphylaxis in young children within the United States.

One of the landmark pediatric studies evaluating mustard allergy was published by Rancé et al., who analyzed 36 children with confirmed mustard allergy [6]. More than half of affected patients developed symptoms before three years of age, supporting the concept that mustard sensitization may occur early in childhood. Clinical manifestations included urticaria, angioedema, and atopic dermatitis, while the reported mean mustard skin prick wheal diameter was approximately 8.8 mm [6]. In comparison, the patient described in this report demonstrated a markedly larger mustard skin prick response measuring a 15 mm wheal and 25 mm flare, suggesting a high degree of sensitization.

Morisset et al. later demonstrated through double-blind placebo-controlled food challenge testing that mustard can provoke clinically significant allergic reactions, including systemic symptoms, in sensitized individuals [5]. Similarly, Figueroa et al. confirmed mustard allergy through food challenge testing and further described associations with mugwort pollen sensitization and cross-reactivity involving plant-derived foods within the Brassicaceae family [7]. Additional work by Poikonen et al. demonstrated shared allergenic 2S albumin proteins between mustard and turnip rape, supporting the role of immunologic cross-reactivity among related plants [8]. The major mustard allergens, sin a 1 and bra j 1, are 2s albumins whose compact, disulfide-stabilized structure confers resistance to heat and digestion, helping explain their persistence in cooked or processed foods [9]. Representative published case reports of mustard-induced allergic reactions and anaphylaxis are summarized in Table 3, highlighting the limited number of reported pediatric cases.

**Table 3:** Selected Published Reports and Studies Describing Mustard Allergy and Mustard-Induced Anaphylaxis

Author, Year	Population	Country	Key Findings
Widstrom and Johansson, 1986 [10]	Young adult female	Sweden	Immunoglobulin E (IgE)-mediated reaction to mustard with recurrent urticaria and angioedema
Vidal et al., 1991 [11]	Middle-aged female	Spain	Mustard-induced anaphylaxis following ingestion
Monreal et al., 1992 [12]	Two adult patients	Spain	Anaphylactic reactions after mustard sauce ingestion
Rance et al., 2000 [6]	36 children	France	Mustard allergy symptoms commonly began before age 3 years; mean skin prick test wheal size 8.8 mm
Morisset et al., 2003 [5]	Mustard-sensitized pediatric patients	France	Double-blind placebo-controlled food challenge confirmed mustard allergy in sensitized children
Figueroa et al., 2005 [7]	Pediatric and adult mustard-allergic patients	Spain	Demonstrated cross-reactivity with mugwort pollen and Brassicaceae family foods
Pałgan et al., 2018 [13]	38-year-old female	Poland	Severe mustard-induced anaphylaxis with urticaria, dyspnea, and hypotension
Otaki et al., 2026 [14]	4-year-old female	Japan	Pediatric mustard-induced anaphylaxis
Present case	23-month-old male	United States	Mustard-induced anaphylaxis with 15 mm wheal and 25 mm flare on skin prick testing

An important aspect of mustard allergy involves geographic differences in food labeling regulations and public awareness. European studies consistently recognize mustard as a clinically important food allergen in both adults and children, and mustard is included among mandatory labeled allergens in the European Union and Canada [4-8]. In contrast, mustard is not currently included among allergens requiring mandatory disclosure under FALCPA in the United States. This regulatory gap may contribute to reduced public awareness and increased risk of accidental exposure, particularly given mustard's frequent presence as a hidden ingredient within processed foods and condiments. Notably, sesame was added to the FALCPA major allergen list in 2023 following similar advocacy efforts, providing precedent for expanding the list based on emerging clinical evidence. The present case supports ongoing clinical and regulatory advocacy for the inclusion of mustard among mandatory labeled allergens in the United States.

The present case additionally highlights the limited published literature regarding mustard-induced anaphylaxis in U.S. toddlers. Although pediatric mustard allergy has been described extensively in European cohorts, comparable North American pediatric data remain sparse. Several factors strongly supported mustard as the causative allergen in this patient, including isolated mustard exposure, prior tolerance to accompanying foods, rapid onset of mucocutaneous and respiratory symptoms, prompt response to epinephrine, and strong confirmatory skin prick testing. Serum mustard-specific IgE testing was deferred given the unambiguous clinical presentation and is planned at future follow-up. An oral food challenge was not pursued given the severity of the index reaction; the clinical presentation independently satisfied established NIAID/FAAN diagnostic criteria for anaphylaxis [2,3].

This case reinforces the importance of considering mustard allergy in toddlers presenting with food-triggered anaphylaxis, particularly when exposure involves condiments or processed foods. Early recognition, confirmatory allergy testing, strict avoidance counseling, and prompt epinephrine administration remain essential components of management.

Limitations

Several limitations of this case report merit acknowledgment. Serum mustard-specific IgE was not obtained at the time of initial evaluation, though the clinical and skin prick testing data independently supported the diagnosis. An oral food challenge was not performed given the severity of the presenting reaction. The exposure history was based on parental report, which introduces the possibility of recall bias, though the described clinical course was internally consistent and no alternative allergen exposure was identified. Additionally, the causative product, Heinz yellow mustard, is a multi-ingredient condiment containing distilled white vinegar, mustard seed, water, salt, turmeric, spices, and natural flavor, and the possibility that a co-formulated ingredient contributed to the reaction cannot be entirely excluded; however, the strongly positive mustard skin prick response and absence of other identified culprit foods support mustard seed as the primary allergen [15]. Finally, as a single case report, generalizability is inherently limited, and broader epidemiologic conclusions regarding mustard allergy in U.S. children cannot be drawn from this case alone.

## Conclusions

We report a case of IgE-mediated mustard-induced anaphylaxis in a 23-month-old male baby, representing a rare North American pediatric presentation of this underrecognized food allergen. The diagnosis was supported by a clear and isolated exposure history, prior tolerance of accompanying foods, rapid multi-system symptom onset, prompt response to epinephrine, and markedly positive skin prick testing. This case illustrates that mustard can provoke life-threatening anaphylaxis in very young children in the United States and that clinical suspicion for uncommon allergens must be maintained in toddlers presenting with food-triggered systemic reactions.

Beyond the individual patient, this case carries broader implications for allergen labeling policy. Mustard is not currently required to be disclosed as a major allergen under FALCPA, despite being a mandatory labeled allergen in the European Union and Canada. The addition of sesame to the FALCPA major allergen list in 2023 demonstrates that regulatory expansion is achievable. Accumulating clinical evidence, including cases such as this one, supports a similar advocacy effort for mustard. Additional prospective North American pediatric studies are needed to better define the prevalence, clinical phenotype, and natural history of mustard allergy in U.S. children and to strengthen the evidence base for potential regulatory action.
